# Active Targeted Nanoparticles for Delivery of Poly(ADP-ribose) Polymerase (PARP) Inhibitors: A Preliminary Review

**DOI:** 10.3390/ijms221910319

**Published:** 2021-09-25

**Authors:** Saman Sargazi, Mahwash Mukhtar, Abbas Rahdar, Mahmood Barani, Sadanad Pandey, Ana M. Díez-Pascual

**Affiliations:** 1 Cellular and Molecular Research Center, Research Institute of Cellular and Molecular Sciences in Infectious Diseases, Zahedan 9816743463, Iran; sgz.biomed@gmail.com; 2 Faculty of Pharmacy, Institute of Pharmaceutical Technology and Regulatory Affairs, University of Szeged, Eötvös utca 6, 6720 Szeged, Hungary; mahwash.mukhtar@szte.hu; 3 Department of Physics, Faculty of Science, University of Zabol, Zabol 538-98615, Iran; a.rahdar@uoz.ac.ir; 4 Medical Mycology and Bacteriology Research Center, Kerman University of Medical Sciences, Kerman 7616913555, Iran; mahmoodbarani7@gmail.com; 5 Department of Chemistry, College of Natural Science, Yeungnam University, 280 Daehak-Ro, Gyeongsan 38541, Korea; sadanand.au@gmail.com or; 6 Universidad de Alcalá, Facultad de Ciencias, Departamento de Química Analítica, Química Física e Ingeniería Química, Ctra. Madrid-Barcelona, Km. 33.6, 28805 Alcalá de Henares, Madrid, Spain

**Keywords:** nanotechnology, nanomaterials, DNA repair, Poly(ADP-ribose) polymerases, PARP inhibitors, targeted treatment, drug resistance mechanism, toxicity

## Abstract

Nanotechnology has revolutionized novel drug delivery strategies through establishing nanoscale drug carriers, such as niosomes, liposomes, nanomicelles, dendrimers, polymeric micelles, and nanoparticles (NPs). Owing to their desirable cancer-targeting efficacy and controlled release, these nanotherapeutic modalities are broadly used in clinics to improve the efficacy of small-molecule inhibitors. Poly(ADP-ribose) polymerase (PARP) family members engage in various intracellular processes, including DNA repair, gene transcription, signal transduction, cell cycle regulation, cell division, and antioxidant response. PARP inhibitors are synthetic small-molecules that have emerged as one of the most successful innovative strategies for targeted therapy in cancer cells harboring mutations in DNA repair genes. Despite these advances, drug resistance and unwanted side effects are two significant drawbacks to using PARP inhibitors in the clinic. Recently, the development of practical nanotechnology-based drug delivery systems has tremendously improved the efficacy of PARP inhibitors. NPs can specifically accumulate in the leaky vasculature of the tumor and cancer cells and release the chemotherapeutic moiety in the tumor microenvironment. On the contrary, NPs are usually unable to permeate across the body’s normal organs and tissues; hence the toxicity is zero to none. NPs can modify the release of encapsulated drugs based on the composition of the coating substance. Delivering PARP inhibitors without modulation often leads to the toxic effect; therefore, a delivery vehicle is essential to encapsulate them. Various nanocarriers have been exploited to deliver PARP inhibitors in different cancers. Through this review, we hope to cast light on the most innovative advances in applying PARP inhibitors for therapeutic purposes.

## 1. Introduction

Despite recent progress in cancer research, this multifactorial disease is responsible for the death of millions of men and women worldwide, and many challenges must still be tackled to increase patient survival [1,2]. In this respect, in the last decade, much effort has been made to discover novel and effective therapies that can alleviate undesirable effects induced by radiotherapy or conventional chemotherapeutic agents [1,3,4]. The emergence of multidrug resistance (MDR) is another major obstacle towards the targeted delivery of such therapeutics to malignant cells [5]. Different technologies have already been introduced to clinical practice to overcome these barriers or are presently being investigated in clinical trials [1,6].

Over the past few years, there have been tremendous efforts to develop novel new carriers for various cancer treatments [7,8,9,10,11,12,13,14,15,16]. The advent of nanotechnology and machine learning have helped to design novel alternative targeting strategies to circumvent MDR [17,18,19,20]. As an innovative field with immense potential, nanomedicine allowed biocompatible materials to be developed for various theranostic applications [1,21,22]. Having ushered in multiple established drug delivery platforms, nanostructures such as niosomes [23], liposomes [24], nanomicelles [25], polymeric micelles [26], and nanoparticles (NPs) [27,28,29,30] were broadly used in clinics to enhance the efficacy of anticancer agents for single and combinatorial treatments. Due to their specific design, structural variety, pH-sensitivity, excellent stability, biocompatibility, high drug loading, and simple elaboration, these nano-sized materials have attracted much attention as a new reversal MDR tool in cancer therapy [31,32].

Small-molecule inhibitors have revolutionized the treatment of cancer [33], and autoimmune [34], infectious [35], and metabolic [36,37] diseases. These selective inhibitors can effectively target a wide range of signaling pathways in cancerous cells, such as protein tyrosine kinases (PTKs) and protein tyrosine phosphatases (PTPs) [38], mitogen-activated protein kinase (MAPK) pathway [39], vascular endothelial growth factor (VEGF), epidermal growth factor (EGF) and their receptors (VEGFR and EGFR) [40,41], hedgehog signaling pathway [42], the activator of transcription-3 (Stat3) signaling pathway [43], phosphoinositide 3-kinase (PI3K)/Akt and the mammalian target of Rapamycin signaling network [44,45], Wnt/beta-catenin signaling [46], transforming growth factor β (TGFB) signaling [47], insulinlike growth factor I receptor signaling [48], and DNA repair pathways [49,50,51,52].

The targeting of DNA repair pathways is among the different strategies to combat MDR [53]. In this context, the Poly(ADP-ribose) polymerase (PARP) family members are known to engage in various biological and cellular processes, such as DNA repair, gene transcription, signaling cascades, regulation of the cell cycle, cell division, and intracellular antioxidant response [54,55]. PARP inhibitors account for one of the most remarkable novel strategies for targeted therapy against cancer cells [56]. These synthetic small-molecules act through synthetic lethality in cancer cells having mutations in DNA repair genes [57]. Some of the PARP inhibitors have already been approved to treat cancers with germline mutations in the BRCA1 and BRCA2 genes [58]. At the same time, druggable genomic changes are varied and include a minority of patients with a specific cancer type, limiting the examination of the efficacy of these inhibitors in clinical trials [2].

Drug resistance and unwanted side effects are two significant drawbacks to using PARP inhibitors for cancer therapy [50,56]. Therefore, new formulations containing these selective inhibitors were subsequently designed to overcome MDR. Through this review, we hope to cast light on the most innovative progress made in applying PARP inhibitors for therapeutic purposes.

## 2. PARP Inhibitors

### 2.1. Classification

There PARP family is comprised of 17 members out of which the primary nuclear PARPs are of Poly(ADP-ribose) polymerase-1 (PARP-1), Poly(ADP-ribose) polymerase-2 (PARP-2), Poly(ADP-ribose) polymerase-3 (PARP-3), PARP-5a, PARP5b, and tankyrase 1 and 2 [54]. The small-molecules including olaparib (AZD-2281, TOPARP-A), veliparib (ABT-888), talazoparib (BMN-673), rucaparib (AG014699, PF-01367338, CO338), niraparib (MK4827), BMN 763, AZD2461 (NCT01247168), E7016 (NCT01127178), INO-1001 (NCT00272415), EP9722 (NCT00920595) are potent submicromolar competitive nicotinamide adenine dinucleotide (NAD+) inhibitors of PARP-1 and PARP-2 enzymes [59,60]. Inhibition of PARP enzymes blocks PARylation reaction, through which ADP-ribose residues transfer to target substrates via ADP-ribosyl transferase using NAD+ [61]. It has been established that PARP trapping is responsible for the anticancer potency of PARP inhibitors [62]. Among all the PARP inhibitors in clinical development, talazoparib is the most potent PARP inhibitor, whereas veliparib demonstrated the lowest PARP trapping potency [60]. With less inhibitory effect than against PARP-1 and PARP-2, olaparib and rucaparib can also inhibit PARP-3 in BRCA-mutated advanced cancers [61].

### 2.2. Molecular Mechanism of Action and Role of NPs

Endogenous and exogenous DNA damaging agents cause cellular stresses that result in DNA damage [63]. These damages pose a threat to the genome and are routinely repaired by different mechanisms, such as base excision repair (BER), nucleotide excision repair (NER), mismatch repair (MMR), ataxia-telangiectasia mutated/ataxia-telangiectasia, and Rad3-related (ATM/ATR)-mediated DNA damage response, nonhomologous end-joining repair (NHEJ) and more importantly, homologous recombination (HR) pathways [64,65]. On the other hand, PARP enzymes contribute to these mechanisms by acting as proteins that share enzymatic and scaffolding activities and have broad roles in overall DNA repair mechanisms [54].

**Figure 1:**
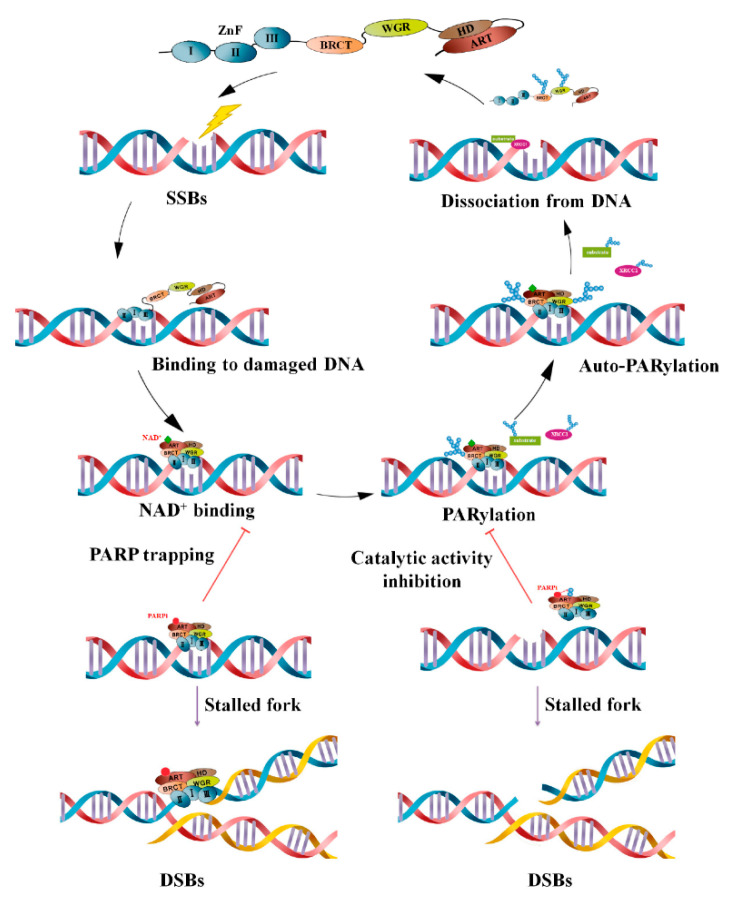
Mechanisms of PARP-mediated PARylation following the occurrence of a single-strand break (SSB). PARP enzymes (specifically PARP-1) are promptly recruited to the damaged site with the help of a zinc finger domain. By using NAD+ as substrate, PARylation enhances the recruitment of DNA damage proteins, including X-ray repair cross complementing group 1 (XRCC1), DNA ligase III, etc. Auto-PARylation of PARP diminishes its affinity for DNA, and PARP enzymes dissociate from DNA. As competitive inhibitors, PAPR inhibitors could bind to the pocket instead of NAD+, a situation generally referred to as PARP trapping. PARPs trapping at the replication fork leads to aggregation of unrepaired SSBs and double-strand breaks (DSBs) and ultimately induces cell death in cancer cells. Reprinted from ref. [61].

PARP1 consists of three domains that are involved in auto-modification, PARylation (catalysis) and DNA-binding. In case of single-strand break (SSB) or double-strand break (DSB) in DNA, PARP1 recruits to the damaged site and forms branched poly (ADP-ribose) (PAR) chains. Other PARPs, including PARP2, PARP5a, and PARP5b, create long branching PAR chains of up to 200 units in length [66]. The formed chain would protect DNA from nuclease enzymes and facilitates the recruitment of DNA repair proteins involved in BER, HR, and NHEJ pathways [54,67]. Later, the poly (ADP-ribose) glycohydrolase (PARG) enzyme effectively hydrolyses poly ADP-ribose units [68]. PARP inhibitors entrap PARP enzymes and destabilize replication forks at the damaged site of DNA [69]. This results in inducing apoptotic cell death via replication-stress mitotic catastrophe, and therefore suppresses tumor growth via suppressing the DNA damage repair pathway [70,71]. In addition to PARP inhibitors, PARG inhibitors can exacerbate replication deficiencies and be considered promising therapeutic modalities against cancer types with genomic instability [69]. Figure 1 demonstrates mechanisms of PARP-mediated PARylation following DNA damage.

NPs serve as a unique platform of drug delivery and therefore they have been extensively investigated for their potential use in anticancer drug delivery. NPs can be fabricated in a variety of ways to increase the drug encapsulation capacity at the inner core, and they can be also equipped with multiple functions on the outer core to improve the drug activity in the target environment [72]. Besides, they have the potential to deliver poorly water-soluble drugs and provide a sustained releasing profile to prolong the blood circulation time. Thus, NPs offer far superior pharmacokinetics compared to small molecule drugs [73]. Many promising drugs fail to pass clinical trials due to their short half-life and high toxicity in vivo. Besides, orally administered drugs undergo extensive degradation in the liver resulting in decreased optimum concentration of the drugs before reaching the target site. However, if the drugs could be loaded in specially designed NPs for delivery, the drugs would circulate for longer times in the blood, enabling sustained interaction with the tumor and leading to increased tumor accumulation. NPs also serve as a sheath that would shield the body from off-target toxicities of drugs, alter the cellular uptake of the drugs and lessen the probability of the emergence of drug resistance. At present, many NPs are being studied in clinical trials for a wide variety of medical treatments, and a few of them have been clinically approved for chemotherapies [74]. For example, conventional oral delivery of PARP inhibitors is hindered by limited bioavailability and off-target toxicities [75]. On the other hand, due to complementary activity of PARP inhibitors, the use of them during radiotherapy (RT) has yielded promising results. Unfortunately, this approach is often hindered by toxicity and poor in vivo stability of the PARP inhibitors [76]. Additionally, the preclinical PARP inhibitors are limited by their rapid washout kinetics and consequently modest pharmacological performances [77]. In several cases, these could be improved by loading the PARP into nanoparticulates, improving blood half-life, in vivo uptake and overall pharmacodynamics [77,78]. For instance, olaparib has advanced the treatment of ovarian cancer by providing patients with an effective and molecularly targeted maintenance therapy. However, olaparib must undergo first-pass metabolism. A nanoparticle delivery system has the advantage of administering olaparib directly into the peritoneal cavity for local treatment [79].

### 2.3. Clinical Efficacy

PARP inhibitors were introduced as tools to protect from inflammatory diseases [80]. Later, these selective inhibitors were evaluated as nanotherapeutic agents in clinical trials as targeted treatment strategies against solid tumors derived from ovarian, prostate, breast, colorectal, and uterine tissues [54,81]. Although previous reports have established that PARP inhibitors effectively treat BRCA1-deficient cancers and increase patients’ progression-free survival (PFS), new studies have suggested that HR-deficient cells may also be vulnerable to PARP inhibition [82]. Moreover, PARP inhibitors in combination with conventional chemotherapeutics or radiation have shown promising results in treating different cancers [83,84]. We have previously shown that a combination of AZD2461 and valproic acid, a histone deacetylase inhibitor, activates apoptotic cell death in phosphatase and tensin homolog (PTEN)-deficient prostate cancer cells [85]. A phase II preclinical study showed that rucaparib, combined with temozolomide, increased the PFS in patients with invasive melanoma [86]. In a cohort study, de Bono et al. suggested olaparib to treat metastatic castration-resistant prostate cancer in patients who have alterations in HR-related genes [80]. Despite their efficacy in cancer treatment, some researchers have recently proposed that inhibition of PARP can be considered an innovative approach for treating metabolic disorders, cardiovascular diseases, testicular damage, and nephropathies [87,88,89,90]. More recently, Fritz et al. introduced emerging roles for PARP inhibitors in hematological cancers, particularly acute leukemia [91].

## 3. Nanoformulations for Delivery of PARP Inhibitors to Cancer Cells

Nanotechnology remains a novel platform for cancer therapeutics. Nanomediated drug and protein delivery have been widely investigated in cancer treatment [92,93,94,95]. There are certain limitations to the delivery of chemotherapeutics, such as encapsulation of anticancer moiety, low water solubility, immediate drug release, short circulation life, and low safety index. All these obstacles in cancer therapeutics can be easily resolved by using nanotechnology [96]. Additionally, the choice of suitable nanovehicles and their core composition greatly impacts pharmacokinetics and rational therapy. Recently, there has been an increased significance of delivering PARP inhibitors using nanotechnology for cancer therapeutics. Few such delivery vehicles have been studied and are being presented in this review.

### 3.1. Liposomes

Nanotechnology has made tremendous advancements in the treatment of cancer. Liposomal drug delivery holds potential in nanotherapeutics. The characteristics of liposomes closely resemble the biological membrane and improve the permeation of both hydrophilic and hydrophobic drugs. The leaky vasculature in the tumor and cancers allow the accumulation of liposomes and hence the activity. A unique drug delivery system conjugated with BMN 673 (talazoparib) was designed based on the successful phase I clinical trial related to PARP inhibitor and cisplatin. BMN 673 is a PARP inhibitor and exhibits antitumor activity. BMN 673 has presented safety and efficacy in phase I and phase II clinical trials in both breast cancer and ovarian cancer. Cisplatin was conjugated with BMN 673 and encapsulated in the liposomal vehicle developed with a layer-by-layer approach. Moreover, HA was coated onto the liposomal vehicle to target CD44 receptors, overexpressed in ovarian cancer. The poly(L-Lysine) (PLL) constituent modulated the release of the drug at tumor pH. The drug and BMN 673 conjugated liposomal delivery system improved the tumor treatment in the mice [97]. BMN-673 was also used in another study, and the comparison was conducted with the nanoformulations containing olaparib to inhibit the DNA gene repair. The injectable nanoformulations demonstrated that BMN-673 was a more attractive choice as it inhibited the colony formation as a monotherapy in an efficient way. Moreover, BMN-673 nanoformulation has an improved bioavailability profile as compared to olaparib nanoformulation [98].

Talazoparib was encapsulated in the liposomes in another approach for the BRCA-mutated metastatic breast cancer therapy. As talazoparib suffers limitations such as anemia, thrombocytopenia, and reduced bioavailability, it was incorporated into the nanodrug delivery system. Liposomes were synthesized using the layer-by-layer technique. The nanoliposomes were incorporated with talazoparib and were investigated for immunomodulation in the BRCA-deficient mice [75]. There was a significant reduction in off-site toxicity in the BRCA-deficient mice, leading to reduced signs of thrombocytopenia. Furthermore, the survival time of the BRCA-deficient mice was improved on treatment with the liposomes. DNA damage was induced in the tumor cells and inhibited their proliferation.

Liposomes improve the delivery of more than one drug simultaneously, which otherwise poses therapeutic issues. This is possible due to enhanced pharmacokinetics, solubility, and tissue distribution of the liposomes. The FDA-approved olaparib is the PARP inhibitor for ovarian cancer and a breakthrough therapy status for metastatic castration-resistant prostate cancer (mCRPC). The anticancer effect of carboplatin and olaparib can be enhanced when used in combination using PEGylated liposomes. Olaparib is the PARP inhibitor that induces DNA damage in cancer cells, and the therapeutic activity can be augmented when combined with a chemotherapeutic agent. The liposomal NPs (OLICARB) were fabricated using the different molar concentrations (1:1 and 2:1) of olaparib and carboplatin. Liposomes were characterized by flameless atomic absorption spectrometry (FAAS). Immunofluorescence analysis presented the damage to DNA in cancerous cells by OLICARB NPs. There was found to be a decrease in the growth of 3D mammospheres by the OLICARB NPs. The antitumor activity of both the molar ratios of OLICARB NPs revealed that the therapy could be an optimistic approach for treating tumors [99].

Radiotherapy has been investigated for many years for the treatment of cancer. Recently, PARP inhibitors have been used in combination with radiotherapy to enhance DNA damage in the tumor cells. Lipid formulation was developed using olaparib (NanoOlaparib) to measure their efficacy in the prostate cancer cell lines. The activity of the NanoOlaparib was investigated along with the focused beam of X-ray radiation in the Pten/Trp53-deficient mouse model. The therapy elevated the DNA damage in the radiation-resistant cells. After 13 weeks of therapy with NanoOlaparib and radiation, the mice’s survival was prolonged. Additionally, the NanoOlaparib accumulated in the cancer cells up to 19 folds. Altogether, NanoOlaparib was found to be the optimistic delivery vehicle for improving the radiosensitivity in prostate cancer [100]. Nanoformulation containing olaparib (NanoOlaparib) and the nanoformulation with olaparib along with platinum conjugation (NnaoOlaparibPt) were formulated and the activity was demonstrated against the ovarian cancer cell line. The nanoformulations improved the pharmacokinetic and bioavailability profile. The cytotoxicity as studied on the ovarian cancer cell line revealed that nanoformulations were able to suppress cell proliferation. NanoOlaparib improved the therapeutic activity by reducing tumor proliferation. An elevated response was shown by using NanoOlaparib and NanoOlaparibPt on the MDR cell line SKOV-3. Additionally, the use of olaparib and cisplatin in the nanoformulation was developed and found to significantly affect cancer cell death [98].

Another lipid-based nanoformulation was designed for the delivery of olaparib. The lipospheres were developed by the melt dispersion and nanosuspension by wet milling and solvent evaporation. The pharmacokinetic profile and hematological toxicity were compared for the olaparib lipospheres and nanosuspension. The drug was released in a controlled and sustained fashion for up to 9 h. The average particle size was found to be in the range of 53.11 to 126.71 nm with high stability. The NPs were distributed in the tissues with high bioavailability and no toxicity. This simple yet innovative strategy can enhance the activity of PARP inhibitors [101]. The liposomes containing doxorubicin and olaparib were developed, and the studies were conducted on the 3D cell screening model, i.e., 3D multicellular tumor spheroids (MCTS). The liposomes were effective when the combination of anticancer drug along with PARP inhibitor was used. The combination therapy improved the drug accumulation in the MCTS, demonstrating a good delivery carrier for ovarian cancer [102].

Chemotherapy often suffers chemoresistance, and therefore, using chemosensitizers along with chemotherapy can improve cancer treatment. Many studies have been conducted on the delivery of chemosensitizers with a broad range of activity for the tumors and no negative impact on normal tissues. Lipid-based structures are the most widely studied vehicles for encapsulating chemosensitizers. In an exciting approach, two chemosensitizers were employed, Pi3 kinase inhibitor (wortmannin) and PARP inhibitor (olaparib). Both the formulations reduced the tumor proliferation and growth in the lung cancer and breast cancer mouse models compared to the native drugs. There were no traces of toxicity in the other organs. So, it can be stated that there was an improvement in the therapeutic index of chemotherapeutics following this approach [103].

Inhibition of DNA-dependent protein kinase (DNA-PK) is responsible for the cytotoxicity in the myeloma cells. The combination therapy using PARP inhibitor and DNA-PK can further improve tumor growth and regression. Olaparib was used in combination with PI-103 DNA-PK inhibitor and encapsulated in the nanocarriers. PI-103 was used in the form of a prodrug, which self-assembled into a phospholipid bilayer. This layer served as the outer coat of the nanocarriers. Olaparib was encapsulated in the core later. The average particle size was found to be 150 nm with high colloidal stability. The drug was released over the time of 24 h in the tumor microenvironment. The lipid NPs exhibited potent cytotoxic activity against the myeloma cells with high significance compared to the monotherapy. More importantly, the new formulation did not affect the viability of the normal cells [104].

Plectin is a protein that is mislocalized on the surface of ovarian cancer cells. Hence, it can be considered as a therapeutic target for active drug delivery in cancer. A study demonstrated this idea by developing the plectin-targeted peptide anchored to the NPs and loaded with an AZ7379 PARP inhibitor. The plectin-targeted peptide conjugated liposomes significantly decreased cell proliferation in the mice bearing OVCAR8 (epithelial ovarian cancer). The findings affirmed the advantage of nanotechnology and active targeting in improving cancer therapeutics [105].

Intraperitoneal liposomal preparations of PARP inhibitors have also been investigated to treat metastatic ovarian cancer. The intraperitoneal administration improved the chemotherapy as compared to intravenous administration. Olaparib was used as a PARP inhibitor and was encapsulated in the lipid NPs. These NanoOlaparib presented high cytotoxicity to the cancer cells (404 tumor cell line) compared to the free olaparib after administration directly into the peritoneal cavity. NanoOlaparib accumulated in the cancer tissues for up to 72 h after a single dose. Altogether, NanoOlaparib reduced tumor growth with no toxicity to the normal cells [79].

### 3.2. Polymeric NPs

Nanomaterials comprised of polymers have high stability, biodegradation, and biocompatibility. The composition of the NPs can modulate the drug release and enhance the targeting of the cancer cells.

#### 3.2.1. Poly-(d,l-Lactide-Co-Glycolide) (PLGA)

The oral delivery of talazoparib has limitations such as organ toxicity and slow release in the cancer tissues. Hence, developing the nanomediated delivery system can facilitate the release at the tumor microenvironment and reduce off-site toxicity. Following this approach, poly-(d,l-lactide-co-glycolide) (PLGA)-based implants were developed with nanopores which modulated the slow and sustained release of PARP inhibitor over 28 days. The drug delivery implant inhibited the tumor growth in the BRCA deficient mice following intratumoral injection. Furthermore, the talazoparib implant demonstrated low weight loss as compared to the free drug. Besides, talazoparib from the implants enhanced the DNA damage and suppressed tumor cells’ proliferation [106]. Olaparib has been thoroughly investigated by using different nanomediated drug delivery vehicles. Combining olaparib with other agents such as proto-oncogenic transcription factors can further improve the treatment of aggressive cancers.

Photodynamic therapy (PDT) has been the most investigated alternate chemotherapy. The use of PDT generates reactive oxygen species (ROS), resulting in cell damage and eventually cancer death [107]. PDT can be merged with nanotechnology for cancer therapeutics by encapsulating the photosensitizers in the nanocarriers. The NPs have high circulation life and are prone to accumulate in cancer cells with leaky vasculature. Alongside, the toxicity resulting from traditional chemotherapy can be reduced by following this approach. In the current research, methylene blue was coloaded with veliparib in the PLGA NPs. The average particle size was found to be 90 nm with high colloidal stability. The use of PLGA modulated the release of the encapsulated moieties to a controlled behavior. The simultaneous encapsulation of the PARP inhibitor and methylene blue improved the efficacy of the treatment. The photoactivity was found to increase by this encapsulation approach. Overall, the results demonstrated that the cytotoxicity was enhanced by using PDT therapy combined with PARP inhibitor [108].

One such strategy uses Forkhead Box M1 (FOXM1) small interfering RNA FOXM1-siRNA to silicate tumorigenesis genes. FOXM1 is highly expressed in triple-negative breast cancer cells (TNBCs) and hence can be explored as a target. By keeping this rationale in mind, olaparib, along with FOXM1-siRNA, was incorporated into a nanoformulation. PLGA NPs coated with chitosan were developed and loaded with a PARP inhibitor in combination with FOXM1-siRNA. The NPs were successfully taken up by the MDA-MB-231 breast cancer cells and presented cytotoxicity. Altogether, the polymeric NPs were optimistic in the suppression of cancer cell proliferation [109].

#### 3.2.2. Methoxy Poly(ethylene glycol)-poly(ε-caprolactone) (MPEG-PCL)

Similarly, other polymers, such as methoxy poly (ethylene glycol)-poly (e-caprolactone) (MPEG-PCL), have also been utilized in the delivery of olaparib. MPEG-PCL has unique micellar properties that can encapsulate the hydrophilic and hydrophobic drugs together. Human non-small-cell lung cancer cells (NSCLCs) mice were targeted by employing MPEG-PCL and loaded with olaparib. The NPs demonstrated photosensitization followed by radiotherapy. The average particle size of the NPs was estimated to be 31.96 nm. MPEG-PCL NPs loaded with olaparib exhibited an improvement in the survival time of mice and inhibited tumor growth. Additionally, there was a significant increase in the sensitization by using the MPEG-PCL NPs loaded with olaparib compared to free olaparib [110].

#### 3.2.3. Poly(ε-caprolactone)-poly(ethylene glycol)-poly(ε-caprolactone) (PCEC)

Active targeting has also been utilized for delivering PARP inhibitors in cancer therapeutics. Folate receptors are overexpressed in the various tumors and cancer cells, and therefore, developing the folate conjugated NPs can be beneficial in cancer. The use of olaparib improves the radiosensitivity of lung cancer xenografts in the cervical cancer model. Poly (ε-caprolactone)-poly (ethylene glycol)-poly (ε-caprolactone) (PCEC) was used to develop the NPs and were anchored with folate to deliver olaparib. These PCEC NPs were investigated for antitumor activity combined with radiotherapy in the cervical cancer xenografts model. There was found to be enhanced apoptosis and antitumor activity along with high cytotoxicity, as shown by the apoptosis study, immunohistochemical assay, and MTT assay. There was also pronounced DNA damage because of the use of PARP inhibitor [111]. Talazoparib polymeric nanofromulation was developed to determine the activity in the tumor cells. NanoTalazoparib (NanoTLZ) was administered by IV injection in the BRCA deficient mice compared with the free talazoparib. The life span was significantly improved after the IV administration of NanoTLZ and reduced the tumor growth up to 50% of original volume. Moreover, the therapy also maintained the weight, and there was no side effect such as weight loss observed for the nanoformulation. Additionally, there was no off-site adverse effect in the BRCA deficient mice with improved immunomodulation [112].

#### 3.2.4. Pluronic F127

**Figure 2:**
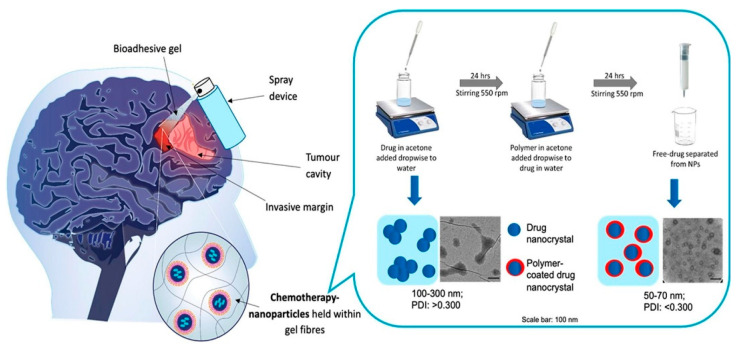
A diagrammatic representation of delivering a PARP inhibitor to glioblastoma cells using a bioadhesive sprayable gel. Reprinted from ref. [113]. under a Creative Commons license (CC BY-NC-ND 4.0).

A very innovative drug delivery system was developed by using polymeric vehicles. Bioadhesive hydrogel, pectin, and drug nanocrystals were coated with polylactic acid-polyethylene glycol to be administered by spray device into the brain parenchyma for treatment of glioblastoma. The NPs exhibited gelling at the calcium concentration in the brain. PARP inhibitor olaparib and the drug etoposide were loaded synergistically in the polymeric vehicle, and pluronic F127 was used during spray-drying. The drug was released over the time of 120 h. The fluorescent imaging revealed that the NPs were accumulated in the mammalian brain. The sprayable hydrogel presented a novel therapeutic platform for malignant brain tumors [113]. Figure 2 represents the process of delivering a PARP inhibitor to glioblastoma cells using a bioadhesive sprayable gel.

### 3.3. Hybrid Nanosystems

The hybrid nanomediated delivery approaches have also been exploited in various diseases. The hybrid system usually comprises more than one component different in physical characteristics but acts in synergy to improve pharmacokinetics [114]. The hybrid system usually uses a polymer and a lipid component or different biopolymers with unique properties. A few examples are cited here for a better understanding.

#### 3.3.1. Superparamagnetic Iron Oxide (IO)—Hyaluronic Acid (HA)

**Figure 3:**
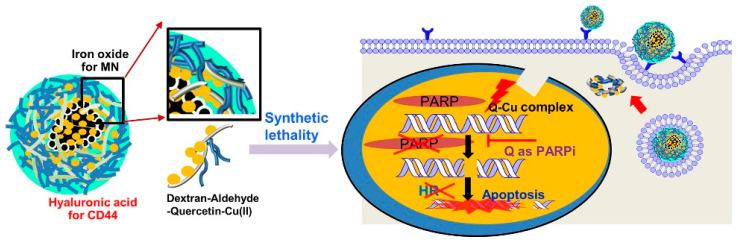
Graphical representation of CuQDA/IO@HA NPs for triple-negative breast cancer therapy. Reprinted with permission from ref. [115]. Copyright 2021 Elsevier, under the license number 5138830604274.

Although the different synthetic PARP inhibitors have been investigated using nanotechnology, there are concerns over the composition of compounds, including safety in the long run. Therefore, many natural ingredients have also been studied as PARP and PARG inhibitors, and investigation is underway for improved cancer therapeutics. In one interesting work, quercetin (Q) was used as a PARP inhibitor of natural origin. Q induces the cleavage of cellular DNA via the formation of metal ions, but it suffers low solubility and poor bioavailability. To overcome this therapeutic hurdle, it was used in conjugation with dextran-aldehyde (DA), and later Cu(II) was incorporated with the previously developed amphiphilic QDA to form the CuQDA. The use of Cu(II) enhanced the DNA cleavage role. To further improve the targeted delivery, CD44 receptor targeting and magnetic navigation were employed by using HA and superparamagnetic iron oxide (IO), respectively. The conjugated system is hence comprised of CuQDA/IO@HA NPs with improved tumor selectivity. Physicochemical characterization presented the optimal size distribution of NPs. The CuQDA/IO@HA NPs posed toxicity to the BRCA-mutant cancer cells in the in vitro studies and were biocompatible to the normal cells. The animal studies revealed that the median survival of the BRCA-mutant mice was prolonged to 61 days compared to 34 days with unmodified Q treatment. This hybrid nanosystem was an exemplary study that conjoined the natural elements with metallic ions to improve cancer cell targeting [115] (Figure 3).

#### 3.3.2. Metal-Organic Frameworks (MOFs)—PEG

The latest approach used metal-organic frameworks (MOFs) as hybrid materials to deliver PARP inhibitors. MOFs are the hybrid structures of metal ions and organic ligands that can also be merged with nanotechnology. The nano-based MOFs (nMOFs) can be exploited in oncology for increasing the radiation dose. nMOFs induces reactive oxygen species upon irradiation and leads to cancer cell damage. A novel nMOFs was fabricated that demonstrated DNA damage in the malignant cells. These nMOFs comprised of high-Z element Hf and the ligand 1,4-dicarboxybenzene (Hf-BDC) and were encapsulated with PARP inhibitors, talazoparib, and buparlisib, in conjugation with PEG (TB@Hf-BDC-PEG). TB@Hf-BDC-PEG elevated DNA damage on irradiation. The therapy presented no toxicity and was beneficial over the conventional MOFs [76].

### 3.4. Self-Assembled NPs

Likewise, self-assembled nanostructures have been exploited for the delivery of PARP inhibitors.

#### 3.4.1. Amphiphilic Peptides

**Figure 4:**
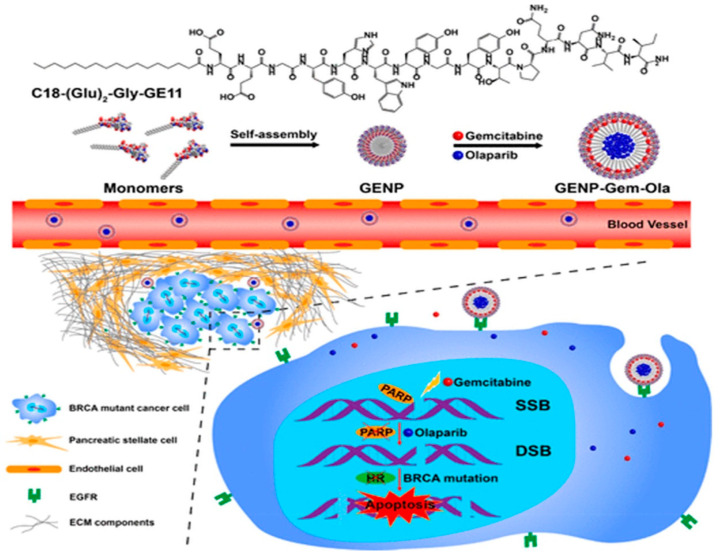
The schematic mechanism of EGFR-targeting self-assembled NPs for codelivery of olaparib and gemcitabine in the BRCA mutant pancreatic cancer. Reprinted with permission from ref. [116]. Copyright 2018 American Chemical Society.

The amphiphilic peptides are capable of self-assembly that can efficiently incorporate PARP inhibitors for effective cancer therapy. To address the BRCA mutations in pancreatic cancer, olaparib was delivered in conjugation with gemcitabine. The codelivery of the PARP inhibitor along with the drug demonstrated synergistic activity in the tumor cells. GE11 peptide was self-assembled to form an amphiphilic nanoparticle (GENP) to treat BRCA mutant pancreatic cancer. Moreover, GENP was functionalized with EGFR for improving tumor targeting. These self-assembled GENPs improved the pharmacokinetic profile as well as the accumulation of drugs in pancreatic cancer cells. There was a significant increase in the tumor growth in the murine model of pancreatic cancer [116] (Figure 4).

#### 3.4.2. Poloxamer Micelles

Though PARP inhibitors have shown favorable efficacy in cancer treatment, most of the compounds suffer solubility issues. Nevertheless, different delivery strategies have been developed, and the most promising are the micellar formulations and self-assembled systems. Talazoparib along with Pi3 kinase inhibitor (buparlisib), were incorporated together in the mixed poloxamer micelle (MPM) formulation. MPM improved the administration of both the agents and resulted in proficient breast cancer suppression in the cancer model. Moreover, there was a significant elevation in the radiosensitivity with the codelivery of talazoparib and buparlisib. No toxicity was exhibited in the normal tissues. The biodistribution studies were found to be promising and resulted in reducing tumor growth followed by DNA damage and apoptosis of malignant cells [117].

#### 3.4.3. Tannic Acid-Docetaxel Self-Assemblies (DSAs)

Previously docetaxel has been exploited in the treatment of prostate cancer. However, recently a self-assembled TA and docetaxel-based nanosystem was fabricated. TA was used as a PARP inhibitor and to improve the chemosensitization. The nanoformulation of tannic acid-docetaxel self-assemblies (DSAs) demonstrated high antitumor activity as compared to free docetaxel. The chemotherapy stress usually induces cellular senescence and is exhibited by high β-galactosidase activity. DSAs improved the chemotherapy, reduced the tumor growth, and regulated the cellular senescence by modulation of TGFβR1/FOXO1/p21 signaling, as shown by immunoblot analysis. DSAs reduced β-galactosidase levels and induced apoptotic cell death in the pancreatic cell xenograft mouse model. The therapy improved the delivery and targeting of docetaxel and resulted in regression of the tumor [118].

### 3.5. Novel Nanosystems

#### 3.5.1. Protein-Based Nanovehicle

TNBCs have the highest tendency of recurrence, and therefore, there is an urgent need to develop targeted therapy with low cytotoxicity [119]. Although PARP inhibitors have been previously investigated, there is an ongoing trend of using novel delivery carriers. One such finding focused on the use of olaparib by improving its bioavailability profile [120]. Olaparib suffers low bioavailability due to the high expression of MDR in TNBCs [121]. Therefore, a protein-based nanovehicle was fabricated using ferritin H-chain (HFn). HFn has an affinity for the transferrin receptor-1 (TfR1) that is overexpressed in TNBCs. Thus, the main purpose was to utilize the concept of active targeting without the anchorage of extra ligand on the surface of the NPs. 24-mer of HFn self-assembled to form the nanocargoes, showing high stability and unique surface topology. This HFn olaparib nanoformulation (HOla) was assessed for the anticancer activity on the non-mutated and BCRA-mutated TNBCs. HOla exhibited high cytotoxicity (1000-fold) as compared to free olaparib. Altogether, HOla mediated the cleavage of PARP-1 along with improved delivery of olaparib to the TNBCs and proved an optimistic nanodelivery vehicle.

#### 3.5.2. Betacaryophyllene (BCP) Carrier

The natural constituent of copaiba oil, betacaryophyllene (BCP), has been investigated to be antioxidant and anticancer. BCP was used as a PARP inhibitor delivery vehicle to improve the antitumor activity. BCP was used because of its good solubility and permeation profile. The research focused on the use of nanoemulsion of BCP as a carrier of talazoparib. Talazoparib has low bioavailability that can be improved by developing parenteral nanoemulsion. Talazoparib was used along with BCP as an oil phase, and polysorbate 80 was used as an emulsifier. The fluorescent imaging revealed the higher cellular uptake of the talazoparib in the cancer cells. Apoptosis and cytotoxicity studies using CellTiter^®^ Blue were found to be concentration-dependent with the sustained release of talazoparib in the different cancer cell lines [122].

#### 3.5.3. Lipids and Cholesterol Nanoemulsion

**Figure 5:**
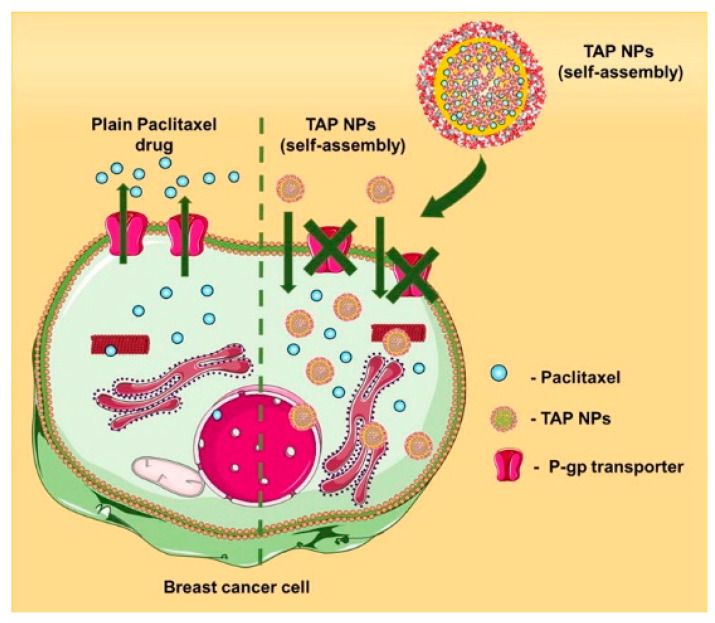
Diagrammatic illustration of the self-assembled tannic acid nanocarriers loaded with paclitaxel with reduced P-glycoproteins efflux pump activity. Reprinted with permission from ref. [123]. Copyright 2019 Elsevier, under the license number 5139510913463.

Certain phenolic constituents of plants possess chemosensitization property along with anticancer property. One such compound is tannic acid (TA) which also exhibits PARP inhibition characteristics. Surprisingly, due to its excellent solubility and biocompatibility, TA can also serve as a nanocarrier that can incorporate various compounds. Paclitaxel has long been used as a chemotherapeutic drug in different cancer types, including breast cancer. Previously, no such formulation was developed in which paclitaxel was synergistically administered with PARP inhibitor/chemosensitizer. Nanoformulation was produced by a simple self-assembly technique, which demonstrated an average particle size of 102 nm. The intracellular uptake of the nanoformulation in the MDA-MB-231 cells was found to be constant over the time of 6 h with 95.52% of release of paclitaxel. The therapeutic profile of the developed TA NPs loaded with paclitaxel was determined by Western blot and microarray analysis, and the results were promising. Additionally, the TA NPs induced apoptosis in the breast cancer cells, followed by β-tubulin stabilization. Additionally, the MDR-mediated drug efflux was reduced in the case of paclitaxel-loaded TA NPs compared to the free paclitaxel [123]. Figure 5 shows a schematic illustration of the abovementioned study.

Another novel molecule, fluorescent PARP inhibitor (PARPi-FL), a fluorescently labeled sensor of olaparib was studied to target the cancer cells. Nanoformulation was developed, which encapsulated the PARPi-FL. The encapsulation not only improved the delivery of the agent to the cancer cells but also helped in the imaging [124]. The nanoemulsion was stabilized with lipids and cholesterol. The nanoemulsion improved permeation followed by subsequent uptake by the PARP1-expressing small cell lung cancer (SCLC). The PARPi-FL nanoemulsion was tested in the xenograft mouse models of SCLC and exhibited good circulation. This nanoemulsion presented good imaging and targeting possibilities [77].

#### 3.5.4. Nano-SiO_2_

Forthwith, silicon dioxide with nanodimension (nano-SiO_2_) is gaining attention in chemotherapeutics. Nano-SiO_2_ exhibits certain toxic effects on the DNA, and thus, this property might be exploited to achieve useful results. However, extensive safety evaluations of nano-SiO_2_ still have not been performed. So, the toxicity concerns should be kept in mind while developing the nano-SiO_2_ based systems. One finding was focused on the inhibition of PARP-1 (mRNA expression) by using nano-SiO_2_. The results demonstrated the reduced PARP-1 expression with a subsequent increase in PARP-1 methylation. The impact of epigenetic modification on this PARP-1 reduction by nano-SiO_2_ was studied using the human epidermal keratinocyte cell line (HaCaT) with or without DNA methyltransferase 1 (DNMT1). The HaCaT cell line was incubated with the nano-SiO_2_ and treated with a DNMT inhibitor to evaluate the epigenetic modification. The outcomes following the real-time Q-PCR and Western blotting showed that methylation of PARP-1 was the major event reasonable for the reduced PARP-1 expression after incubation with nano-SiO_2_ [125].

##### 3.5.5. (IV) BZP NPs

A novel compound 1-(5-(3-(benzofuran-2-yl)-1-phenyl-1H-pyrazol-4-yl)-4,5-dihydro-3-(1H-pyrrol-2-yl)pyrazol-1-yl)ethanone (IV) BZP was encapsulated in the NPs and evaluated for the anticancer activity against breast cancer cell line. (IV) BZP is a benzofuran-pyrazole derivative that exhibits cytotoxicity against several cell lines. In the study in question, the cell lines MCF-7 and MDA-MB-231 were used to compare the activity of (IV) BZP and (IV) BZP NPs. No toxicity was posed to the normal cells. (IV)BZP NPs significantly improved the cytotoxicity against the MCF-7 and MDA-MB-231 cell lines. Apoptosis was also confirmed by the increase in caspase-3 level and downregulation of Bcl-2 protein expression [126]. Categorization of NP-based delivery of PARP inhibitors for the treatment of different cancer types is summarized in Table 1.

## 4. Conclusions and Outlook

PARP inhibition has opened windows of opportunity to treat many diseases, specifically solid tumors. Yet, drug resistance and unwanted side effects are two significant drawbacks to using them for therapeutic purposes. These selective inhibitors have been widely explored by formulating nanomedicine to reduce off-site toxicity or drug resistance. The NPs loaded with PARP inhibitors have shown significant improvement in cancer therapeutics. In addition, PARP inhibitors can be explored as diagnostic therapy along with targeted delivery in cancers. There is still a gap between the laboratory findings and clinical translation of these developed nanoformulations. Further investigations on the tumor microenvironment and MDR mechanisms are needed to minimize or eliminate the limitations of using these inhibitors. An extensive effort needs to be put into exploring nanoformulations in terms of their safety, non-specific accumulation, tissue targeting, and efficacy.

## Figures and Tables

**Table 1:** Categorization of NP-based delivery of PARP inhibitors for the treatment of different cancer types.

Nanocarrier	Type	Refs
Liposomes	Phospholipids	[75,79,97,98,99,100,101,102,103,104,105]
Polymeric NPs	Poly-(d,l-lactide-co-glycolide) (PLGA)	[106,108]
	Methoxy poly (ethylene glycol)-poly (e-caprolac-304 tone) (MPEG-PCL)	[110]
	Poly (ε-caprolactone)-poly (ethyleneglycol)-poly (ε-caprolactone) (PCEC)	[111]
	Pluronic F127	[113]
Hybrid Nanosystems	Quercetin (Q), dextran-aldehyde (DA), Cu(II) and superparamagnetic iron oxide (IO)	[115]
	Metal-organic frameworks (MOFs) and PEG	[76]
Self-Assembled NPs	Amphiphilic peptides	[116]
	Poloxamer micelle (MPM)	[117]
	Tannic acid-docetaxel self-assemblies	[118]
Novel Nanosystems	Protein-based nanovehicle	[121]
	Betacaryophyllene (BCP) carrier	[122]
	Lipids and cholesterol nanoemulsion	[77]
	Nano-SiO_2_	[125]

## Data Availability

Data are included within this article.

## References

[1] Pucci C., Martinelli C., Ciofani G. (2019). Innovative approaches for cancer treatment: Current perspectives and new challenges. Ecancermedicalscience.

[2] Zugazagoitia J., Guedes C., Ponce S., Ferrer I., Molina-Pinelo S., Paz-Ares L. (2016). Current challenges in cancer treatment. Clin. Ther..

[3] Nurgali K., Jagoe R.T., Abalo R. (2018). Adverse effects of cancer chemotherapy: Anything new to improve tolerance and reduce sequelae?. Front. Pharmacol..

[4] Zhang Q.-Y., Wang F.-X., Jia K.-K., Kong L.-D. (2018). Natural product interventions for chemotherapy and radiotherapy-induced side effects. Front. Pharmacol..

[5] Makovec T. (2019). Cisplatin and beyond: Molecular mechanisms of action and drug resistance development in cancer chemotherapy. Radiol. Oncol..

[6] Rosenblum D., Joshi N., Tao W., Karp J.M., Peer D. (2018). Progress and challenges towards targeted delivery of cancer therapeutics. Nat. Commun..

[7] Aferni A.E., Guettari M., Tajouri T., Rahdar A. (2020). The confinement of PVP in AOT microemulsions: Effect of water content and PVP concentration regime on electrical percolation phenomenon. J. Mol. Liq..

[8] Arshad R., Pal K., Sabir F., Rahdar A., Bilal M., Shahnaz G., Kyzas G.Z. (2021). A review of the nanomaterials use for the diagnosis and therapy of salmonella typhi. J. Mol. Struct..

[9] Hakami T.M., Davarpanah A., Rahdar A., Barrett S. (2018). Structural and magnetic study and cytotoxicity evaluation of tetra-metallic nanoparticles of Co0. 5Ni0. 5CrxFe2-xO4 prepared by co-precipitation. J. Mol. Struct..

[10] Hasanein P., Rahdar A., Bahabadi S.E., Kumar A., Kyzas G.Z. (2021). Manganese/cerium nanoferrites: Synthesis and toxicological effects by intraperitoneal administration in rats. Inorg. Chem. Commun..

[11] Heydari M., Yousefi A.R., Rahdar A., Nikfarjam N., Jamshidi K., Bilal M., Taboada P. (2021). Microemulsions of tribenuron-methyl using Pluronic F127: Physico-chemical characterization and efficiency on wheat weed. J. Mol. Liq..

[12] Mohammadi L., Pal K., Bilal M., Rahdar A., Fytianos G., Kyzas G.Z. (2021). Green nanoparticles to treat patients from Malaria disease: An overview. J. Mol. Struct..

[13] Nikazar S., Sivasankarapillai V.S., Rahdar A., Gasmi S., Anumol P., Shanavas M.S. (2020). Revisiting the cytotoxicity of quantum dots: An in-depth overview. Biophys. Rev..

[14] Rahdar A., Aliahmad M., Samani M., HeidariMajd M., Susan M.A.B.H. (2019). Synthesis and characterization of highly efficacious Fe-doped ceria nanoparticles for cytotoxic and antifungal activity. Ceramics Int..

[15] Zou Q., Xing P., Wei L., Liu B. (2019). Gene2vec: Gene subsequence embedding for prediction of mammalian N6-methyladenosine sites from mRNA. RNA.

[16] Yang Y., Liu J., Zhou X. (2021). A CRISPR-based and Post-amplification Coupled SARS-CoV-2 Detection with a Portable Evanescent Wave Biosensor. Biosens. Bioelectron..

[17] Gao Z., Zhang L., Sun Y. (2012). Nanotechnology applied to overcome tumor drug resistance. J. Control. Release.

[18] Wang X.-F., Gao P., Liu Y.-F., Li H.-F., Lu F. (2020). Predicting thermophilic proteins by machine learning. Curr. Bioinform..

[19] Niu M., Lin Y., Zou Q. (2021). sgRNACNN: Identifying sgRNA on-target activity in four crops using ensembles of convolutional neural networks. Plant Mol. Biol..

[20] Sun S., Xu L., Zou Q., Wang G. (2021). BP4RNAseq: A babysitter package for retrospective and newly generated RNA-seq data analyses using both alignment-based and alignment-free quantification method. Bioinform..

[21] Sheervalilou R., Shirvaliloo M., Sargazi S., Ghaznavi H. (2021). Recent advances in iron oxide nanoparticles for brain cancer theranostics: From in vitro to clinical applications. Exp. Opin. Drug Deliv..

[22] Shirvalilou S., Khoei S., Esfahani A.J., Kamali M., Shirvaliloo M., Sheervalilou R., Mirzaghavami P. (2021). Magnetic Hyperthermia as an adjuvant cancer therapy in combination with radiotherapy versus radiotherapy alone for recurrent/progressive glioblastoma: A systematic review. J. Neurooncol..

[23] Tila D., Yazdani-Arazi S.N., Ghanbarzadeh S., Arami S., Pourmoazzen Z. (2015). pH-sensitive, polymer modified, plasma stable niosomes: Promising carriers for anti-cancer drugs. EXCLI J..

[24] Chang D.-K., Chiu C.-Y., Kuo S.-Y., Lin W.-C., Lo A., Wang Y.-P., Li P.-C., Wu H.-C. (2009). Antiangiogenic targeting liposomes increase therapeutic efficacy for solid tumors. J. Biol. Chem..

[25] Raveendran R., Bhuvaneshwar G., Sharma C.P. (2016). Hemocompatible curcumin–dextran micelles as pH sensitive pro-drugs for enhanced therapeutic efficacy in cancer cells. Carbohydr. Polym..

[26] Biswas S., Kumari P., Lakhani P.M., Ghosh B. (2016). Recent advances in polymeric micelles for anti-cancer drug delivery. European J. Pharm. Sci..

[27] Cong Y., Wang L., Wang Z., He S., Zhou D., Jing X., Huang Y. (2016). Enhancing therapeutic efficacy of cisplatin by blocking DNA damage repair. ACS Med. Chem. Lett..

[28] Hosseinikhah S.M., Barani M., Rahdar A., Madry H., Arshad R., Mohammadzadeh V., Cucchiarini M. (2021). Nanomaterials for the Diagnosis and Treatment of Inflammatory Arthritis. Int. J. Mol. Sci..

[29] Miri A., Sarani M., Khatami M. (2020). Nickel-doped cerium oxide nanoparticles: Biosynthesis, cytotoxicity and UV protection studies. RSC Adv..

[30] Nazaripour E., Mousazadeh F., Moghadam M.D., Najafi K., Borhani F., Sarani M., Ghasemi M., Rahdar A., Iravani S., Khatami M. (2021). Biosynthesis of lead oxide and cerium oxide nanoparticles and their cytotoxic activities against colon cancer cell line. Inorg. Chem. Commun..

[31] Farooq M.A., Aquib M., Farooq A., Haleem Khan D., Joelle Maviah M.B., Sied Filli M., Kesse S., Boakye-Yiadom K.O., Mavlyanova R., Parveen A. (2019). Recent progress in nanotechnology-based novel drug delivery systems in designing of cisplatin for cancer therapy: An overview. Artif. Cells Nanomed. Biotechnol..

[32] Panzarini E., Dini L. (2014). Nanomaterial-induced autophagy: A new reversal MDR tool in cancer therapy?. Mol. Pharm..

[33] Wang M., Liu Y., Cheng Y., Wei Y., Wei X. (2019). Immune checkpoint blockade and its combination therapy with small-molecule inhibitors for cancer treatment. Biochim. Biophys. Acta Rev. Cancer.

[34] Prieto-Peña D., Dasgupta B. (2020). Biologic agents and small-molecule inhibitors in systemic autoimmune conditions: An update. Pol. Arch. Intern. Med..

[35] Smithgall T.E., Thomas G. (2013). Small molecule inhibitors of the HIV-1 virulence factor, Nef. Drug Disc. Today Technol..

[36] Watanabe M., Uesugi M. (2013). Small-molecule inhibitors of SREBP activation–potential for new treatment of metabolic disorders. MedChemComm.

[37] Kannt A., Rajagopal S., Kadnur S.V., Suresh J., Bhamidipati R.K., Swaminathan S., Hallur M.S., Kristam R., Elvert R., Czech J. (2018). A small molecule inhibitor of Nicotinamide N-methyltransferase for the treatment of metabolic disorders. Sci. Rep..

[38] Jiang Z.-X., Zhang Z.-Y. (2008). Targeting PTPs with small molecule inhibitors in cancer treatment. Cancer Metastasis Rev..

[39] Yap J.L., Worlikar S., MacKerell A.D., Shapiro P., Fletcher S. (2011). Small Molecule Inhibitors of the ERK Signalling Pathway: Towards Novel Anti-Cancer Therapeutics. ChemMedChem.

[40] Ivy S.P., Wick J.Y., Kaufman B.M. (2009). An overview of small-molecule inhibitors of VEGFR signaling. Nat. Rev. Clin. Oncol..

[41] Zhong H., Phillip Bowen J. (2011). Recent advances in small molecule inhibitors of VEGFR and EGFR signaling pathways. Curr. Top. Med. Chem..

[42] Peukert S., Miller-Moslin K. (2010). Small-molecule inhibitors of the hedgehog signaling pathway as cancer therapeutics. ChemMedChem: Chem. Enabling Drug Discov..

[43] Deng J., Grande F., Neamati N. (2007). Small molecule inhibitors of Stat3 signaling pathway. Curr. Cancer Drug Targ..

[44] McNamara C.R., Degterev A. (2011). Small-molecule inhibitors of the PI3K signaling network. Future Med. Chem..

[45] Segerström L., Baryawno N., Sveinbjörnsson B., Wickström M., Elfman L., Kogner P., Johnsen J.I. (2011). Effects of small molecule inhibitors of PI3K/Akt/mTOR signaling on neuroblastoma growth in vitro and in vivo. Int. J. Cancer.

[46] Voronkov A., Krauss S. (2013). Wnt/beta-catenin signaling and small molecule inhibitors. Curr. Pharm. Des..

[47] Akhurst R.J. (2006). Large-and small-molecule inhibitors of transforming growth factor-ß signaling. Curr. Opin. Investig. Drugs.

[48] Gable K.L., Maddux B.A., Penaranda C., Zavodovskaya M., Campbell M.J., Lobo M., Robinson L., Schow S., Kerner J.A., Goldfine I.D. (2006). Diarylureas are small-molecule inhibitors of insulin-like growth factor I receptor signaling and breast cancer cell growth. Mol. Cancer Ther..

[49] Srinivasan A., Gold B. (2012). Small-molecule inhibitors of DNA damage-repair pathways: An approach to overcome tumor resistance to alkylating anticancer drugs. Future Med. Chem..

[50] Steffen J.D., Brody J.R., Armen R.S., Pascal J.M. (2013). Structural implications for selective targeting of PARPs. Front. Oncol..

[51] Xue C., You J., Zhang H., Xiong S., Yin T., Huang Q. (2021). Capacity of myofibrillar protein to adsorb characteristic fishy-odor compounds: Effects of concentration, temperature, ionic strength, pH and yeast glucan addition. Food Chem..

[52] Xu L., Jiang S., Wu J., Zou Q. (2021). An in silico approach to identification, categorization and prediction of nucleic acid binding proteins. Briefings Bioinform..

[53] Sadeghi M.B., Nakhaee A., Saravani R., Sargazi S. (2021). Significant association of LXRβ (NR1H2) polymorphisms (rs28514894, rs2303044) with type 2 diabetes mellitus and laboratory characteristics. J. Diabetes Metab. Disord..

[54] Anwar M., Aslam H.M., Anwar S. (2015). PARP inhibitors. Hered. Cancer Clin. Pract..

[55] Martínez-Romero R., Martínez-Lara E., Aguilar-Quesada R., Peralta A., Oliver F.J., Siles E. (2008). PARP-1 modulates deferoxamine-induced HIF-1α accumulation through the regulation of nitric oxide and oxidative stress. J. Cell. Biochem..

[56] Patel M., Nowsheen S., Maraboyina S., Xia F. (2020). The role of poly (ADP-ribose) polymerase inhibitors in the treatment of cancer and methods to overcome resistance: A review. Cell Biosci..

[57] Dedes K.J., Wilkerson P.M., Wetterskog D., Weigelt B., Ashworth A., Reis-Filho J.S. (2011). Synthetic lethality of PARP inhibition in cancers lacking BRCA1 and BRCA2 mutations. Cell Cycle.

[58] Azim H.A., Kassem L., Azim H. (2020). Integrating PARP inhibitors into the management of breast cancer: Where are we?. Chin. Clin. Oncol..

[59] Bogliolo S., Cassani C., Dominoni M., Musacchi V., Venturini P.L., Spinillo A., Ferrero S., Gardella B. (2016). Veliparib for the treatment of ovarian cancer. Expert Opin. Investig. Drugs.

[60] Murai J., Pommier Y. (2015). Classification of PARP inhibitors based on PARP trapping and catalytic inhibition, and rationale for combinations with topoisomerase I inhibitors and alkylating agents. PARP Inhibitors for Cancer Therapy.

[61] Min A., Im S.-A. (2020). PARP inhibitors as therapeutics: Beyond modulation of PARylation. Cancers.

[62] Hopkins T.A., Shi Y., Rodriguez L.E., Solomon L.R., Donawho C.K., DiGiammarino E.L., Panchal S.C., Wilsbacher J.L., Gao W., Olson A.M. (2015). Mechanistic dissection of PARP1 trapping and the impact on in vivo tolerability and efficacy of PARP inhibitors. Mol. Cancer Res..

[63] Mehta I.S., Kulashreshtha M., Chakraborty S., Kolthur-Seetharam U., Rao B.J. (2013). Chromosome territories reposition during DNA damage-repair response. Genome Biol..

[64] Yan S., Sorrell M., Berman Z. (2014). Functional interplay between ATM/ATR-mediated DNA damage response and DNA repair pathways in oxidative stress. Cell. Mol. Life Sci..

[65] Guo H., Liu H., Wu H., Cui H., Fang J., Zuo Z., Deng J., Li Y., Wang X., Zhao L. (2019). Nickel carcinogenesis mechanism: DNA damage. Int. J. Mol. Sci..

[66] Qi H., Price B.D., Day T.A. (2019). Multiple roles for mono-and poly (ADP-ribose) in regulating stress responses. Trends Genet..

[67] Wei H., Yu X. (2016). Functions of PARylation in DNA damage repair pathways. Genom. Proteom. Bioinform..

[68] Houl J.H., Ye Z., Brosey C.A., Balapiti-Modarage L.P., Namjoshi S., Bacolla A., Laverty D., Walker B.L., Pourfarjam Y., Warden L.S. (2019). Selective small molecule PARG inhibitor causes replication fork stalling and cancer cell death. Nat. Commun..

[69] Slade D. (2020). PARP and PARG inhibitors in cancer treatment. Genes Dev..

[70] Virág L., Szabó C. (2002). The therapeutic potential of poly (ADP-ribose) polymerase inhibitors. Pharmacol. Rev..

[71] Farmer H., McCabe N., Lord C.J., Tutt A.N., Johnson D.A., Richardson T.B., Santarosa M., Dillon K.J., Hickson I., Knights C. (2005). Targeting the DNA repair defect in BRCA mutant cells as a therapeutic strategy. Nature.

[72] Amreddy N., Babu A., Muralidharan R., Panneerselvam J., Srivastava A., Ahmed R., Mehta M., Munshi A., Ramesh R. (2018). Recent advances in nanoparticle-based cancer drug and gene delivery. Adv. Cancer Res..

[73] Amreddy N., Babu A., Muralidharan R., Munshi A., Ramesh R. (2017). Polymeric nanoparticle-mediated gene delivery for lung cancer treatment. Polym. Gene Delivery Syst..

[74] Ventola C. (2017). LProgress in Nanomedicine: Approved and Investigational Nanodrugs. Pharm. Ther..

[75] Di Zhang P.B., Leal A.S., Carapellucci S., Sridhar S., Liby K.T. (2019). A nano-liposome formulation of the PARP inhibitor Talazoparib enhances treatment efficacy and modulates immune cell populations in mammary tumors of BRCA-deficient mice. Theranostics.

[76] Neufeld M.J., DuRoss A.N., Landry M.R., Winter H., Goforth A.M., Sun C. (2019). Co-delivery of PARP and PI3K inhibitors by nanoscale metal–organic frameworks for enhanced tumor chemoradiation. Nano Res..

[77] Gonzales J., Kossatz S., Roberts S., Pirovano G., Brand C., Pérez-Medina C., Donabedian P., de la Cruz M.J., Mulder W.J., Reiner T. (2018). Nanoemulsion-based delivery of fluorescent PARP inhibitors in mouse models of small cell lung cancer. Bioconjug. Chem..

[78] Singh B., Yang S., Krishna A., Sridhar S. (2020). Nanoparticle formulations of poly (ADP-ribose) polymerase inhibitors for cancer therapy. Front. Chem..

[79] Baldwin P., Ohman A.W., Tangutoori S., Dinulescu D.M., Sridhar S. (2018). Intraperitoneal delivery of NanoOlaparib for disseminated late-stage cancer treatment. Int. J. Nanomed..

[80] De Bono J., Mateo J., Fizazi K., Saad F., Shore N., Sandhu S., Chi K.N., Sartor O., Agarwal N., Olmos D. (2020). Olaparib for metastatic castration-resistant prostate cancer. N. Engl. J. Med..

[81] Majera D., Skrott Z., Bouchal J., Bartkova J., Simkova D., Gachechiladze M., Steigerova J., Kurfurstova D., Gursky J., Korinkova G. (2019). Targeting genotoxic and proteotoxic stress-response pathways in human prostate cancer by clinically available PARP inhibitors, vorinostat and disulfiram. Prostate.

[82] Setton J.S., Powell S.N. (2020). Moving beyond PARP Inhibition in ATM-Deficient Prostate Cancer. Cancer Res..

[83] Murai J., Zhang Y., Morris J., Ji J., Takeda S., Doroshow J.H., Pommier Y. (2014). Rationale for poly (ADP-ribose) polymerase (PARP) inhibitors in combination therapy with camptothecins or temozolomide based on PARP trapping versus catalytic inhibition. J. Pharmacol. Exp. Ther..

[84] Löser D.A., Shibata A., Shibata A.K., Woodbine L.J., Jeggo P.A., Chalmers A.J. (2010). Sensitization to radiation and alkylating agents by inhibitors of poly (ADP-ribose) polymerase is enhanced in cells deficient in DNA double-strand break repair. Mol. Cancer Ther..

[85] Sargazi S., Saravani R., Reza J.Z., Jaliani H.Z., Mirinejad S., Rezaei Z., Zarei S. (2019). Induction of apoptosis and modulation of homologous recombination DNA repair pathway in prostate cancer cells by the combination of AZD2461 and valproic acid. EXCLI J..

[86] Plummer R., Lorigan P., Steven N., Scott L., Middleton M.R., Wilson R.H., Mulligan E., Curtin N., Wang D., Dewji R. (2013). A phase II study of the potent PARP inhibitor, Rucaparib (PF-01367338, AG014699), with temozolomide in patients with metastatic melanoma demonstrating evidence of chemopotentiation. Cancer Chemother. Pharmacol..

[87] Wasyluk W., Zwolak A. (2021). PARP Inhibitors: An Innovative Approach to the Treatment of Inflammation and Metabolic Disorders in Sepsis. J. Inflamm. Res..

[88] Henning R.J., Bourgeois M., Harbison R.D. (2018). Poly (ADP-ribose) polymerase (PARP) and PARP inhibitors: Mechanisms of action and role in cardiovascular disorders. Cardiovasc. Toxicol..

[89] Celik-Ozenci C., Kuscu N., Gungor-Ordueri N., Tasatargil A., Sahin P., Durmus H. (2017). Inhibition of poly (ADP-ribose) polymerase may have preventive potential for varicocoele-associated testicular damage in rats. Andrology.

[90] Zakaria E.M., El-Maraghy N.N., Ahmed A.F., Ali A.A., El-Bassossy H.M. (2017). PARP inhibition ameliorates nephropathy in an animal model of type 2 diabetes: Focus on oxidative stress, inflammation, and fibrosis. Naunyn-Schmiedeberg’s Arch. Pharmacol..

[91] Fritz C., Portwood S.M., Przespolewski A., Wang E.S. (2021). PARP goes the weasel! Emerging role of PARP inhibitors in acute leukemias. Blood Rev..

[92] Revia R.A., Zhang M. (2016). Magnetite nanoparticles for cancer diagnosis, treatment, and treatment monitoring: Recent advances. Mater. Today.

[93] Rezvantalab S., Drude N.I., Moraveji M.K., Güvener N., Koons E.K., Shi Y., Lammers T., Kiessling F. (2018). PLGA-based nanoparticles in cancer treatment. Front. Pharmacol..

[94] Praetorius N.P., Mandal T.K. (2007). Engineered nanoparticles in cancer therapy. Recent Patents on Drug Delivery & Formulation.

[95] Lohcharoenkal W., Wang L., Chen Y.C., Rojanasakul Y. (2014). Protein nanoparticles as drug delivery carriers for cancer therapy. BioMed. Res. Int..

[96] Awasthi R., Roseblade A., Hansbro P.M., Rathbone M.J., Dua K., Bebawy M. (2018). Nanoparticles in cancer treatment: Opportunities and obstacles. Curr. Drug Targets.

[97] Mensah L.B., Morton S.W., Li J., Xiao H., Quadir M.A., Elias K.M., Penn E., Richson A.K., Ghoroghchian P.P., Liu J. (2019). Layer-by-layer nanoparticles for novel delivery of cisplatin and PARP inhibitors for platinum-based drug resistance therapy in ovarian cancer. Bioeng. Transl. Med..

[98] Baldwin P., Tangutoori S., Sridhar S. (2018). In vitro analysis of PARP inhibitor nanoformulations. Int. J. Nanomed..

[99] Novohradsky V., Zajac J., Vrana O., Kasparkova J., Brabec V. (2018). Simultaneous delivery of olaparib and carboplatin in PEGylated liposomes imparts this drug combination hypersensitivity and selectivity for breast tumor cells. Oncotarget.

[100] van de Ven A.L., Tangutoori S., Baldwin P., Qiao J., Gharagouzloo C., Seitzer N., Clohessy J.G., Makrigiorgos G.M., Cormack R., Pandolfi P.P. (2017). Nanoformulation of olaparib amplifies PARP inhibition and sensitizes PTEN/TP53-deficient prostate cancer to radiation. Mol. Cancer Ther..

[101] Pathade A.D., Kommineni N., Bulbake U., Thummar M.M., Samanthula G., Khan W. (2019). Preparation and comparison of oral bioavailability for different nano-formulations of olaparib. AAPS PharmSciTech.

[102] Eetezadi S. (2016). Nanomedicines and Combination Therapy of Doxorubicin and Olaparib for Treatment of Ovarian Cancer. Ph.D. Thesis.

[103] Caster J.M., Sethi M., Kowalczyk S., Wang E., Tian X., Hyder S.N., Wagner K.T., Zhang Y.-A., Kapadia C., Au K.M. (2015). Nanoparticle delivery of chemosensitizers improve chemotherapy efficacy without incurring additional toxicity. Nanoscale.

[104] Patel P., Misra S., Rodriguez N.S., Vulugundam G., Oh A.L., Senyuk V., Mahmud N., Rondelli D., Pan D. (2017). Combined nanoparticle delivery of PARP and DNA-PK inhibition for multiple myeloma. Blood.

[105] Dasa S.S.K., Diakova G., Suzuki R., Mills A.M., Gutknecht M.F., Klibanov A.L., Slack-Davis J.K., Kelly K.A. (2018). Plectin-targeted liposomes enhance the therapeutic efficacy of a PARP inhibitor in the treatment of ovarian cancer. Theranostics.

[106] Belz J.E., Kumar R., Baldwin P., Ojo N.C., Leal A.S., Royce D.B., Zhang D., van de Ven A.L., Liby K.T., Sridhar S. (2017). Sustained release talazoparib implants for localized treatment of BRCA1-deficient breast cancer. Theranostics.

[107] Liu R., Gao Y., Liu N., Suo Y. (2020). Nanoparticles Loading Porphyrin Sensitizers in Improvement of Photodynamic Therapy for Ovarian Cancer. Photodiagnosis Photodyn. Ther..

[108] Magalhães J.A., Arruda D.C., Baptista M.S., Tada D.B. (2021). Co-Encapsulation of Methylene Blue and PARP-Inhibitor into Poly (Lactic-Co-Glycolic Acid) Nanoparticles for Enhanced PDT of Cancer. Nanomaterials.

[109] Misra R., Patra B., Varadharaj S., Verma R.S. (2021). Establishing the promising role of novel combination of triple therapeutics delivery using polymeric nanoparticles for Triple negative breast cancer therapy. BioImpacts: BI.

[110] Wu M., Liu J., Hu C., Li D., Yang J., Wu Z., Yang L., Chen Y., Fu S., Wu J. (2018). Olaparib nanoparticles potentiated radiosensitization effects on lung cancer. Int. J. Nanomed..

[111] Li D., Hu C., Yang J., Liao Y., Chen Y., Fu S.Z., Wu J.B. (2020). Enhanced Anti-Cancer Effect of Folate-Conjugated Olaparib Nanoparticles Combined with Radiotherapy in Cervical Carcinoma. Int. J. Nanomed..

[112] Zhang D., Baldwin P., Sridhar S., Liby K. (2018). Nanoformulated Talazoparib enhances the efficacy and reduces the toxicity of this PARP inhibitor in a preClin. model of BRCA-deficient breast cancer. FASEB J..

[113] McCrorie P., Mistry J., Taresco V., Lovato T., Fay M., Ward I., Ritchie A.A., Clarke P.A., Smith S.J., Marlow M. (2020). Etoposide and olaparib polymer-coated nanoparticles within a bioadhesive sprayable hydrogel for post-surgical localised delivery to brain tumours. Eur. J. Pharm. Biopharm..

[114] Wang Z., Xiang H., Dong P., Zhang T., Lu C., Jin T., Chai K.Y. (2021). Pegylated azelaic acid: Synthesis, tyrosinase inhibitory activity, antibacterial activity and cytotoxic studies. J. Mol. Struct..

[115] Cheng H.-W., Chiang C.-S., Ho H.-Y., Chou S.-H., Lai Y.-H., Shyu W.-C., Chen S.-Y. (2021). Dextran-modified Quercetin-Cu (II)/hyaluronic acid nanoMed. with natural poly (ADP-ribose) polymerase inhibitor and dual targeting for programmed synthetic lethal therapy in triple-negative breast cancer. J. Control. Release.

[116] Du C., Qi Y., Zhang Y., Wang Y., Zhao X., Min H., Han X., Lang J., Qin H., Shi Q. (2018). Epidermal growth factor receptor-targeting peptide nanoparticles simultaneously deliver gemcitabine and olaparib to treat pancreatic cancer with breast cancer 2 (BRCA2) mutation. ACS Nano.

[117] DuRoss A.N., Neufeld M.J., Landry M.R., Rosch J.G., Eaton C.T., Sahay G., Thomas Jr C.R., Sun C. (2019). Micellar formulation of talazoparib and buparlisib for enhanced DNA damage in breast cancer chemoradiotherapy. ACS Appl. Mater. Interfaces.

[118] Nagesh P.K., Chowdhury P., Hatami E., Kumari S., Kashyap V.K., Tripathi M.K., Wagh S., Meibohm B., Chauhan S.C., Jaggi M. (2019). Cross-Linked Polyphenol-Based Drug Nano-Self-Assemblies Engineered to Blockade Prostate Cancer Senescence. ACS Appl. Mater. Interfaces.

[119] Jitariu A.-A., Cîmpean A.M., Ribatti D., Raica M. (2017). Triple negative breast cancer: The kiss of death. Oncotarget.

[120] Mazzucchelli S., Truffi M., Baccarini F., Beretta M., Sorrentino L., Bellini M., Rizzuto M., Ottria R., Ravelli A., Ciuffreda P. (2017). H-Ferritin-nanocaged olaparib: A promising choice for both BRCA-mutated and sporadic triple negative breast cancer. Sci. Rep..

[121] Dufour R., Daumar P., Mounetou E., Aubel C., Kwiatkowski F., Abrial C., Vatoux C., Penault-Llorca F., Bamdad M. (2015). BCRP and P-gp relay overexpression in triple negative basal-like breast cancer cell line: A prospective role in resistance to Olaparib. Sci. Rep..

[122] Mehra N.K., Tekmal R.R., Palakurthi S. (2018). Development and evaluation of talazoparib nanoemulsion for systemic therapy of BRCA1-mutant cancer. Anticancer Res..

[123] Chowdhury P., Nagesh P.K., Hatami E., Wagh S., Dan N., Tripathi M.K., Khan S., Hafeez B.B., Meibohm B., Chauhan S.C. (2019). Tannic acid-inspired paclitaxel nanoparticles for enhanced anticancer effects in breast cancer cells. J. Colloid Interface Sci..

[124] Jing X., Wang H., Huang X., Chen Z., Zhu J., Wang X. (2021). Digital image colorimetry detection of carbaryl in food samples based on liquid phase microextraction coupled with a microfluidic thread-based analytical device. Food Chem..

[125] Gong C., Tao G., Yang L., Liu J., Liu Q., Li W., Zhuang Z. (2012). Methylation of PARP-1 promoter involved in the regulation of nano-SiO2-induced decrease of PARP-1 mRNA expression. Toxicol. Lett..

[126] Anwar M.M., El-Karim A., Somaia S., Mahmoud A.H., Amr A.E.-G.E., Al-Omar M.A. (2019). A Comparative Study of the Anticancer Activity and PARP-1 Inhibiting Effect of Benzofuran–Pyrazole Scaffold and Its Nano-Sized Particles in Human Breast Cancer Cells. Molecules.

